# Human Papillomavirus DNA in LEEP Plume

**DOI:** 10.1155/S1064744994000591

**Published:** 1994

**Authors:** Anil K. Sood, Zahra Bahrani-Mostafavi, Jay Stoerker, I. Keith Stone

**Affiliations:** 1 Department of Obstetrics and Gynecology University of Florida College of Medicine P.O. Box 100294 Gainesville FL 32610-0294 USA; 2 Department of Obstetrics and Gynecology Carolinas Medical Center Charlotte NC USA

## Abstract

*Objective:* This study was undertaken to determine the prevalence of human papillomavirus (HPV) in loop electrosurgical excision procedure (LEEP) plumes.

*Methods:* Forty-nine consecutive patients with colposcopic and cytologic evidence of cervical intraepithelial neoplasia (CIN) were tested. Smoke plumes were collected through a filter placed in the suction tubing. DNA was harvested by proteinase K digest of the filters and prepared for polymerase chain reaction (PCR) by L1 consensus primers.

*Results:* Thirty-nine (80%) tissue samples were positive for HPV, with types 6/11 in 4, 16/18 in 19, 31/33/35 in 2, and other types in 6 patients. The tissue sample was inadequate for typing in 8 patients. HPV DNA was detected in 18 (37%) filters.

*Conclusions:* Although the consequences of HPV in LEEP plume are unknown, it would be prudent to adopt stringent control procedures.


Infectious Diseases in Obstetrics and Gynecology 2:167–170 (1994)
© 1994 Wiley-Liss, Inc.

### Human Papillomavirus DNA in LEEP Plume

**Anil K. Sood, Zahra Bahrani-Mostafavi, Jay Stoerker, and I. Keith Stone**

*Department of Obstetrics and Gynecology, University of Florida College of Medicine, Gainesville, FL (A.K.S., I.K.S.), and Department of Obstetrics and Gynecology, Carolinas Medical Center, Charlotte, NC (Z.B.-M., J.S.)*

Abstract

***Objective:*** **This study was undertaken to determine the prevalence of human papillomavirus (HPV) in loop electrosurgical excision procedure (LEEP) plumes.**

***Methods:*** **Forty-nine consecutive patients with colposcopic and cytologic evidence of cervical intraepithelial neoplasia (CIN) were tested. Smoke plumes were collected through a filter placed in the suction tubing. DNA was harvested by proteinase K digest of the filters and prepared for polymerase chain reaction (PCR) by L1 consensus primers.**

***Results:*** **Thirty-nine (80%) tissue samples were positive for HPV, with types 6/11 in 4, 16/18 in 19, 31/33/35 in 2, and other types in 6 patients. The tissue sample was inadequate for typing in 8 patients. HPV DNA was detected in 18 (37%) filters.**

***Conclusions:*** **Although the consequences of HPV in LEEP plume are unknown, it would be prudent to adopt stringent control procedures.** © 1994 Wiley-Liss, Inc.

Key words

Cervical intraepithelial neoplasia, HPV DNA, loop electrosurgical excision

Loop electrosurgical excision procedure (LEEP) is rapidly becoming an attractive way of performing excision biopsies of the cervix. As early as 1981, Cartier et al.[1] began publishing and presenting experience with diathermy loops in the diagnosis and treatment of lesions of the uterine cervix. These techniques were recently introduced in the United States from Great Britain. The use of electrical current to excise dysplastic regions on the cervix results in the generation of a smoke plume. Previous studies have shown conflicting results regarding the presence of human papillomavirus (HPV) DNA in laser plume. Garden et al.[2] demonstrated the presence of HPV DNA in laser vapor samples from plantar warts. Kashima et al.[3] showed the presence of HPV in laser vapor from recurrent respiratory papillomatosis. Recently, Ferenczy et al.[4] demonstrated the presence of HPV in laser vapor from anogenital condylomas. However, Abramson et al.[5] did not detect HPV DNA in the smoke plume from laser vaporization of laryngeal papillomas. No data exist regarding the presence of HPV in LEEP plume. Therefore, we undertook a study to determine the prevalence of HPV in LEEP-generated plumes.

### MATERIALS AND METHODS

### Patient Population

Our patient population consisted of 49 consecutive women enrolled in the study between February 1992 and January 1993 who had a LEEP performed at the University of Florida. All women had either histologic evidence of cervical intraepithelial neoplasia (CIN) II or CIN III or cytologic evidence of CIN II or CIN III. Endocervical curettage was negative in 42 patients. Seven patients

Address correspondence/reprint requests to Dr. Anil K. Sood, Department of Obstetrics and Gynecology, University of Florida, P.O. Box 100294, Gainesville, FL 32610-0294.

Received June 22, 1994
Accepted September 9, 1994

with a positive endocervical curettage had the extent of the lesion defined by colposcopy and were deemed candidates for loop excision.

### Sample Collection

The loop excision was performed in an outpatient clinic at the University of Florida. A commercially available sterile speculum with a smoke evacuation metal cannula welded on the inner surface of the upper blade was used for exposure of the cervix. The evacuation cannula was not in direct contact with the cervical lesion. The colposcope was used to define the lesion. The cervix and vagina were stained with Lugol's solution to define the squamocolumnar junction. The cervix was injected with 1% lidocaine with epinephrine (1:100,000) in a circumferential fashion using a 22-gauge spinal needle. The loop size was based on the size of the lesion and size of the transformation zone. If the lesion or transformation zone was too large for single-loop excision, then the transformation zone was removed in 2 passes. The procedure was performed under a blend of coagulation and cut. The loop was placed lateral to the edge of the lesion and pushed perpendicularly. It was slowly drawn across the lesion to the opposite side and pulled out of the tissue perpendicularly to produce a button-shaped specimen. The bleeding from the cervical wound was controlled using the diathermy ball. Monsel's solution was also applied to the base of the wound to increase the long-term hemostatic effect.

A small (2 mm × 2 mm) biopsy was taken from the LEEP specimen for HPV analysis. The plume of smoke generated by LEEP was evacuated with the Stackhouse smoke-evacuation system (Stackhouse Association, Inc., El Segundo, CA). The smoke was collected through an in-line filter (Filter disc #65651-801) supplied by Baxter, Inc. During each session, a new sterile suction tubing and filter were used. For controls, 2 new in-line filters were exposed to the air in the procedure room without a patient in the room.

### Sample Analysis

The DNA was prepared from tissue samples by digestion with 50 μg/ml proteinase K in a buffer containing 0.01 M Tris (pH 7.8), 0.005 M EDTA, and 0.5% sodium dodecyl sulfate (SDS); (Sigma, St. Louis, MO). The digests were performed at 55°C overnight, and the DNA was extracted with phenol and precipitated with 2 volumes of 100% ethanol. DNA was harvested from the filters by injection of 2 ml of the same digestion cocktail with a disposable 5.0-ml Luer-lock syringe, closing the filter on the opposite side with another syringe, and sealing both with UV-irradiated parafilm for digestion overnight. The digest was recovered in the same syringe after washing the liquid volume back and forth between the 2 syringes through the filter. The digests from the filters were prepared for DNA analysis both by direct heat inactivation at 95°C for 10 min and by phenol extraction. A control series was created by introducing graded amounts of HPV-6 plasmid onto a filter and making recovery as described.

DNA pellets from the ethanol precipitations described above were resuspended in TE buffer (10 mM Tris, pH 7.8, 0.1 mM EDTA) and the concentrations determined by optical density at 260 nm. The samples were amplified by the polymerase chain reaction (PCR) for human restriction fragment length polymorphism (RFLP) KM-19 and HPV L1 consensus as described in previous reports.[6] Briefly, for HPV analysis, oligonucleotide primers MY 09 and MY 11 were used as to amplify the L1 region of HPVs. The detection of HPV DNA was performed using $^{32}$P-labeled oligonucleotide GP1J as described by Gravitt et al.[7] and the labeling of amplified products from reference strains as described by Resnick et al.[8]

The PCR product was purified using a gel filtration column (Stratagene, La Jolla, CA). Double-stranded sequencing of the purified product was performed using the Circumvent TM Thermal Cycle Dideoxy DNA Sequencing Kit (New England Biolabs, Beverly, MA). Primers were the same as those described for the primary PCR. The products from the sequencing reactions were analyzed using a 6% Long Ranger TM gel (AT Biochem, Malvern, PA). The isolates were sequenced in both strands.

### RESULTS

We performed a LEEP biopsy on 49 patients. Table 1 presents the distribution of diagnoses with cytology, colposcopically directed biopsies, and LEEP biopsies. LEEP biopsy demonstrated low-grade lesions in 14 patients, high-grade lesions in

TABLE 1. Cytologic and histologic findings in patients

| Method | Diagnosis[a] Negative | Atypia | LGSIL | HGSIL |
|---|---|---|---|---|
| Pap smear | 3 | 3 | 5 | 38 |
| Colposcopically directed biopsies | 1 | — | 11 | 37 |
| LEEP biopsy | 7 | — | 14 | 28 |

[a]LGSIL, low-grade squamous intraepithelial lesion; HGSIL, high-grade squamous intraepithelial lesion.

TABLE 2. Correlation of tissue samples, filters, and HPV DNA positivity

| HPV DNA | Tissue [No. (%)] | Filter [No. (%)] |
|---|---|---|
| Positive | 39 (79.6) | 18 (36.7) |
| Negative | 10 (20.4) | 31 (63.3) |

28 patients, and no abnormality in 7 patients. The margins on LEEP biopsy were positive in 18 patients.

Table 2 presents the distribution of HPV DNA in tissue and vapor samples. Thirty-nine (80%) of the tissue samples were positive for HPV. The distribution of HPV is noted in Figure 1. HPV was detected in 18 (37%) filters. The 18 HPV-positive filters came from the 39 patients with HPV-positive tissue samples. DNA sequencing was performed on 8 samples, and in all cases the HPV subtype was identical in the tissue and in the filter. The remainder of the filter samples were inadequate for DNA sequencing. Ten tissue samples were negative for HPV DNA. The filters from these 10 cases were also negative for HPV DNA. The remaining 21 negative filter samples were from patients with HPV-positive tissue samples. Both control filters were negative for HPV DNA.

### DISCUSSION

From this study, it is clear that the plume of smoke generated by LEEP may become contaminated by HPV DNA. Previous studies have shown that HPV DNA can be recovered from laser smoke. Garden et al.[2] recovered intact HPV DNA in the plume of smoke generated by $CO_2$ laser treatment. Kashima et al.[3] recovered HPV DNA from 57% of vapor samples generated from $CO_2$ laser treatment for

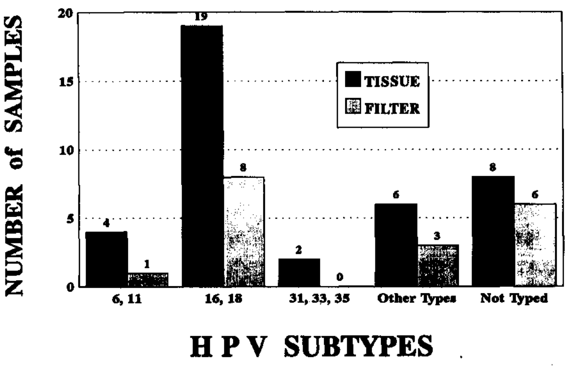

Fig. 1. Distribution of HPV subtypes in tissue and filter samples.

recurrent respiratory papillomatosis. Case studies have shown development of laryngeal papillomatosis with HPV DNA in laser surgeons with no known source of infection other than the surgeon's own patients.[9] However, there are no data regarding the safety of the LEEP plume. In the present study, the prevalence of HPV DNA was high (37% of filters).

PCR is a highly sensitive technique for the detection of HPV DNA. There is concern that the finding of HPV DNA in vapor plume might be the result of contamination; however, the smoke-collection cannula was never in touch with the cervical lesion, and new sterile suction tubing, filter, and speculum were used for each patient. The control filter specimens were negative for HPV. In addition, DNA sequencing confirmed the presence of the same HPV types in both tissue and filter samples.

It is not clear whether the HPV DNA in plume is viable. Studies with the laser have failed to show viability. However, at average power densities, the predominant mode of tissue destruction is very rapid boiling of histologic and cellular water to form steam, which ruptures cells and tissues, ejecting cellular constituents from the crater into the laser beam. There they absorb the direct rays of the laser and are heated to incandescence, thus burning in the presence of oxygen to form a thick, malodorous smoke.[10] Bellina et al.[11] were unable to show any metabolic activity, replication, or transcription in plume collection during $CO_2$ laser treatment of condyloma acuminatum. Mihashi et al.[12] found that the intact airborne cells they recovered from vapor plume failed to grow in tissue culture. Electrosurgery uses low-voltage, high-frequency radio waves through a thin wire loop to accomplish surgical effects. Lateral necrosis or coagulation depends on the current mode, intensity, frequency, impedance, and operating mode. Electrosurgery may cause less tissue destruction than laser and may liberate more intact cells, including viral DNA, which may be more infectious.

Future studies should focus on assessing the viability of cells and DNA in the smoke generated by LEEP. A long-term follow-up of gynecologic surgeons involved with LEEP would also be useful. Although the consequences of HPV in LEEP plume are unknown, it is prudent to reduce the risk of potential infection to the patient, surgeon, and other operating personnel by the use of appropriate gloves and masks and by effective smoke-evacuation methods.

### ACKNOWLEDGMENTS

This work was supported by a research grant from the Southern Medical Association.

### REFERENCES

1. Cartier R, Sopena B, Cartier I: Use of the diathermy loop in the diagnosis and treatment of lesions of the uterine cervix. Colposcopy of the connective tissue. Fourth World Congress, International Federation for Cervical Pathology and Colposcopy, London, 1981.
2. Garden JM, O'Banion MK, Shelnitz LS, et al.: Papillomavirus in the vapor of carbon dioxide laser-treated verrucae. JAMA 259:1199–1202, 1988.
3. Kashima HK, Kessis T, Mounts P, et al.: Polymerase chain reaction identification of human papillomavirus DNA in $CO_2$ laser plume from recurrent respiratory papillomatosis. Otolaryngol Head Neck Surg 104:191–195, 1991.
4. Ferenczy A, Bergeron C, Richart RM: Human papillomavirus DNA in $CO_2$ laser-generated plume of smoke and its consequences to the surgeon. Obstet Gynecol 75: 114–117, 1990.
5. Abramson AL, DiLorenzo TP, Steinberg BM: Is papillomavirus detectable in the plume of laser-treated laryngeal papilloma? Arch Otolaryngol Head Neck Surg 116: 604–607, 1990.
6. Highsmith WE, Perry TR, Prior TW, et al.: Use of the polymerase chain reaction for simultaneous analysis of polymorphisms linked to cystic fibrosis. Clin Chem 35: 1260–1261, 1989.
7. Gravitt PE, Hakenewerth AM, Stoerker J: A direct comparison of methods proposed for widespread screening of human papillomavirus infections. Mol CellProbes 5:152–157, 1991.
8. Resnick RM, Cornelissen MTE, Wright DK, et al.: Detection and typing of human papillomavirus in archival cervical cancer specimens by DNA amplification with consensus primers. J Natl Cancer Inst 82:1477–1484, 1990.
9. Hallmo P, Naess O: Laryngeal papillomatosis with human papillomavirus DNA contracted by a laser surgeon. Eur Arch Otorhinolaryngol 248:425–427, 1991.
10. Wisniewski DM, Warhol MJ, Rando RF, et al.: Studies on the transmission of viral disease via the $CO_2$ laser plume and ejecta. J Reprod Med 35:1117–1123, 1990.
11. Bellina JH, Stiernholm RL, Kurpel SF: Analysis of plume emissions after papilloma irradiation with the carbon dioxide laser. J Reprod Med 27:268–270, 1982.
12. Mihashi S, Jako GJ, Incze J, et al.: Laser surgery in otolaryngology. Interaction of $CO_2$ laser and soft tissue. Ann NY Acad Sci 267:263–294, 1975.
